# Geographical disparities in core population coverage indicators for roll back malaria in Malawi

**DOI:** 10.1186/1475-9276-6-5

**Published:** 2007-07-04

**Authors:** Lawrence N Kazembe, Christopher C Appleton, Immo Kleinschmidt

**Affiliations:** 1 Applied Statistics and Epidemiology Research Unit, Mathematical Sciences Department, Chancellor College, University of Malawi, Zomba, Malawi; 2 School of Biological and Conservation Sciences, University of KwaZulu-Natal, Durban, South Africa; 3 Malaria Research Programme, Medical Research Council, Durban, South Africa

## Abstract

**Background:**

Implementation of known effective interventions would necessitate the reduction of malaria burden by half by the year 2010. Identifying geographical disparities of coverage of these interventions at small area level is useful to inform where greatest scaling-up efforts should be concentrated. They also provide baseline data against which future scaling-up of interventions can be compared. However, population data are not always available at local level. This study applied spatial smoothing methods to generate maps at subdistrict level in Malawi to serve such purposes.

**Methods:**

Data for the following responses from the 2000 Malawi Demographic and Health Survey (DHS) were aggregated at subdistrict level: (1) households possessing at least one bednet; (2) children under 5 years who slept under a bednet the night before the survey; (3) bednets retreated with insecticide within past 6–12 months preceding the survey; (4) children under 5 who had fever two weeks before the survey and received treatment within 24 hours from the onset of fever; and (5) women who received intermittent preventive treatment of malaria during their last pregnancy. Each response was geographically smoothed at subdistrict level by applying conditional autoregressive models using Markov Chain Monte Carlo simulation techniques.

**Results:**

The underlying geographical patterns of coverage of indicators were more clear in the smoothed maps than in the original unsmoothed maps, with relatively high coverage in urban areas than in rural areas for all indicators. The percentage of households possessing at least one bednet was 19% (95% credible interval (CI): 16–21%), with 9% (95% CI: 7–11%) of children sleeping under a net, while 18% (95% CI: 16–19%) of households had retreated their nets within past 12 months prior to the survey. The northern region and lakeshore areas had high bednet coverage, but low usage and re-treatment rates. Coverage rate of children who received antimalarial treatment within 24 hours after onset of fever was consistently low for most parts of the country, with mean coverage of 4.8% (95% CI: 4.5–5.0%). About 48% (95% CI: 47–50%) of women received antimalarial prophylaxis during their pregnancy, with highest rates in the southern and northern areas.

**Conclusion:**

The striking geographical patterns, for example between predominantly urban and rural areas, may reflect spatial differences in provider compliance or coverage, and can partly be explained by socio-economic and cultural differences. The wide gap between high bed net coverage and low retreatment rates may reflect variation in perceptions about malaria, which may be addressed by implementing information, education and communication campaigns or introducing long lasting insecticide nets. Our results demonstrate that DHS data, with appropriate methodology, can provide acceptable estimates at sub-national level for monitoring and evaluation of malaria control goals.

## Background

Disease control programmes, health policy makers and community based organisations need local data for programme monitoring and evaluation and resource allocation [1-5]. This is true for malaria control, where there is an internationally agreed global target to reduce the burden of malaria by half by 2010 [6,7]. A variety of effective personal protective measures have been identified aimed at reducing the burden of disease and include among others, possession, usage and re-treatment of insecticide treated bednets (ITNs), intermittent preventive treatment with antimalarials for pregnant women (IPTp), prompt and effective case management of malaria (PECM) [8-10].

The 2002 malaria policy in Malawi stipulates that by 2006 at least 60% of the population at risk, that is children under five and pregnant women, should sleep under ITNs, and 75% of the nets in use should be retreated within 6–12 months. Similar targets have been set for IPTp and PECM [6]. Governmental and non-governmental organizations (NGOs) have been active in scaling-up these resources to achieve the stipulated goals. The international community partnerships have also started addressing the issue of reducing the burden of malaria in the country by supporting exactly the same interventions [11].

Measuring patterns of coverage for the above-mentioned anti-malarial indicators, as implemented by various health programmes, would be essential not only to highlight areas of low coverage, but also crucial to monitor and evaluate their impact on malaria burden [2,7,12-14]. Interestingly, maps of health indicators are increasingly being used as a way of reporting programme outcomes and impacts or research results to policy makers [4,5]. This approach is easing the presentation and interpretation of results, and is more meaningful to policy makers.

Data for monitoring and evaluating coverage of anti-malarial indicators is mainly population-based household surveys. One of the most common relevant household surveys in malaria-endemic countries is the Demographic and Health Surveys (DHS). The DHS has the advantage of providing reliable, nationally representative estimates. Furthermore, because a standardized methodology is employed, the DHS allows comparisons over time and between countries [13,15]. However, DHS estimates at national level, are too coarse to be meaningful for planning and policy making, monitoring and evaluation, or resource allocation at local level, for instance, at sub-districts. Scaling-up of interventions is often a highly local undertaking, and implementors may be interested in comparing coverage rates across communities within a district where their programmes are being applied. These efforts, however, may fail because of a dearth of relevant and meaningful local data. Using small-area analyses on DHS data and mapping, local or community level estimates can be produced, useful to unmask variations in interventions coverage at these level.

Small area analyses have increased, in the last two decades, with the aim of understanding within-country variations of health indicators, see again Jia *et al*. [1], Petrucci *et al*. [3], Pickle and Su [4], Kleinschmidt *et al*. [5]. Nevertheless, these analyses pose challenges because of small sample sizes and/or that the data may be missing due to the sampling design. Mapping observed proportions at such level, without accounting for sampling noise or sparse distribution of data, may give misleading patterns. Small area techniques, for instance, spatial smoothing techniques can be applied to reduce sampling errors and allow patterns to emerge [1,16,17].

The aim of this study was to map, at subdistrict level in Malawi, geographical disparities of core population coverage indicators for Roll Back Malaria (RBM). The indicators are i) possession of bednets, ii) usage and iii) re-treatment of insecticide treated bednets, iv) prophylaxis with antimalarials for pregnant women, and v) prompt and affective case management of malaria.

## Methods

### Data

The analyses were based on the representative cross-sectional 2000 Malawi DHS household data [15]. The DHS programme has conducted over 190 surveys in about 80 countries globally, almost every 4–5 years, and covers most malaria-endemic countries in Africa [18]. The 2000 Malawi DHS is a third in a series of such surveys carried in the country.

A two-stage stratified sampling design was used to collect the data. At the first stage, enumeration areas (EAs) as defined in the 1998 Malawi Population and Housing Census sample frame were selected stratified by urban-rural designation. A total of 560 EAs: 449 in the rural areas and 111 in the urban areas, were systematically sampled. At the second stage, a fixed number of households were randomly selected in each sampled EA, and all women aged 15–49 years were eligible for interview. A total of 14,213 households were successfully interviewed, representing a household response rate of 99% (98.7% in the urban areas and 99.2% in the rural areas). In the interviewed households, 13,538 eligible women were identified, of which 97.7 percent were interviewed. The household data were obtained through an interviewer-administered questionnaire in the respondent's own language, and information was obtained on a range of health, demographic and socioeconomic topics for each household member. We limited our analysis to the sample of women who had live-births within 5 years preceding the survey. Sampling weights were provided to take account of sampling probability.

All selected EAs were geo-coded, and all household data in EAs were merged to obtain a sub-district sample to which they belong, adjusting for sampling weights accordingly [19]. We aggregated the following key indicators at sub-district level [10]: (1) ownership of ITN, (2) usage of ITN, (3) re-treatment of ITN with insecticide within the past 6–12 months, (4) prompt and effective case management of malaria, and (5) usage of antimalarial preventive treatment by pregnant women. The full definition for each indicator is given in Table 1. Observed proportions of each indicator were calculated in each sub-district and compared by region, and place of residence (rural/urban). The observed proportions for each outcome were mapped at the same level.

**Table 1:** Description of indicators analysed and mapped using 2000 MDHS data

	Indicator	Numerator (*Y*_*i*_)	Denominator (*N*_*i*_)
1.	Ownership of bednets	Number of households possessing at least one bednet	Total number of households surveyed
2.	Use of bednets by under 5 children	Number of under 5 children slept under net night before the survey	Total number of under 5 children in surveyed households
3.	Re-treatment of bednets	Number of households with net retreated with insecticide within past 6–12 months	Total number of households possessing a net
4.	Prompt and effective treatment of fever	Number of under 5 children receiving any anti-malarial treatment within 24 hours of onset of fever	Total number of under 5 children who had fever 2 weeks before survey
5.	Use of intermittent preventive treatment by pregnant women	Number of pregnant women who used antimalarials during their last pregnancy	Total number of pregnant women surveyed who gave birth within 5 years to the survey date

Table 2 shows the number of sub-districts, EAs sampled per region by rural-urban designation, and the mean sample size of households per sub-district by region by residence. The mean sample size of households per subdistrict, in all regions, was 50 (range: 10–150). Those who had fever within 2 weeks preceding the survey were 4245 (41% of total sample), and the mean number per subdistrict was 14 (range: 0–98) children, while those who took IPTp was 5649 (47% of those who had live births), and the mean sample per subdistrict was 38 (range: 4–125). With relatively small samples per sub-district, the challenge is how to obtain stable estimates of coverage indicators, and to allow for relative comparisons with the sub-districts of interest. This problem is circumvented by applying spatial smoothing as described next.

**Table 2:** Sampled sub-districts, enumeration areas (EAs),  and the mean (range) number of households selected per  subdistrict by region by residence, Malawi DHS 2000.

Region	Residence	Subdistricts sampled	Enumeration areas sampled	Households/sub-district Mean (Range)
Northern	Urban	11	20	36 (7–126)
	Rural	28	70	55 (10–246)
Central	Urban	18	36	39 (11–163)
	Rural	71	158	52 (5–156)
Southern	Urban	32	55	31 (6–130)
	Rural	76	221	61 (11–229)

### Spatial data smoothing and mapping

Assume *Y*_*i *_is the number of respondents who owned bednets, used bednets, retreated bednets, received anti-malarials within 24 hours, or received IPTp, out of the total *N*_*i *_sampled in each sub-district *i*. The outcome can be modelled as a binomial response *Y*_*i *_~ *bin*(*p*_*i*_, *N*_*i*_), such that *p*_*i *_is the true coverage proportion of a selected indicator in sub-district *i*. The proportions were smoothed using the following model [16,17],

logit(*p*_*i*_) = α + *u*_*i *_+ *s*_*i*_

where *α *is an intercept, which is interpreted as an overall log-odds coverage for all areas; *u*_*i *_is unstructured heterogeneity effect for subdistrict *i*; *s*_*i *_is a structured spatial component that allows to "borrow information" from adjacent areas, such that neighbouring areas have a similar coverage proportion. By borrowing information from neighbouring sub-districts to supplement their own, even when the sample size is small, creates sufficient statistical power, and generates reliable estimates for comparison of sub-district estimates for evaluation purposes.

This was achieved by assuming a conditional autoregressive (CAR) prior [16], defined as *N*(s¯i|j
 MathType@MTEF@5@5@+=feaafiart1ev1aaatCvAUfKttLearuWrP9MDH5MBPbIqV92AaeXatLxBI9gBaebbnrfifHhDYfgasaacH8akY=wiFfYdH8Gipec8Eeeu0xXdbba9frFj0=OqFfea0dXdd9vqai=hGuQ8kuc9pgc9s8qqaq=dirpe0xb9q8qiLsFr0=vr0=vr0dc8meaabaqaciaacaGaaeqabaqabeGadaaakeaacuWGZbWCgaqeamaaBaaaleaacqWGPbqAcqGG8baFcqWGQbGAaeqaaaaa@3297@,τs2
 MathType@MTEF@5@5@+=feaafiart1ev1aaatCvAUfKttLearuWrP9MDH5MBPbIqV92AaeXatLxBI9gBaebbnrfifHhDYfgasaacH8akY=wiFfYdH8Gipec8Eeeu0xXdbba9frFj0=OqFfea0dXdd9vqai=hGuQ8kuc9pgc9s8qqaq=dirpe0xb9q8qiLsFr0=vr0=vr0dc8meaabaqaciaacaGaaeqabaqabeGadaaakeaaiiGacqWFepaDdaqhaaWcbaGaem4CamhabaGaeGOmaidaaaaa@3106@/*m*_*i*_), where s¯i|j
 MathType@MTEF@5@5@+=feaafiart1ev1aaatCvAUfKttLearuWrP9MDH5MBPbIqV92AaeXatLxBI9gBaebbnrfifHhDYfgasaacH8akY=wiFfYdH8Gipec8Eeeu0xXdbba9frFj0=OqFfea0dXdd9vqai=hGuQ8kuc9pgc9s8qqaq=dirpe0xb9q8qiLsFr0=vr0=vr0dc8meaabaqaciaacaGaaeqabaqabeGadaaakeaacuWGZbWCgaqeamaaBaaaleaacqWGPbqAcqGG8baFcqWGQbGAaeqaaaaa@3297@ is the pooled mean of area *i*, based on the adjacent areas *j*, and *m*_*i *_are the number of sub-districts neighbouring *i*, while τs2
 MathType@MTEF@5@5@+=feaafiart1ev1aaatCvAUfKttLearuWrP9MDH5MBPbIqV92AaeXatLxBI9gBaebbnrfifHhDYfgasaacH8akY=wiFfYdH8Gipec8Eeeu0xXdbba9frFj0=OqFfea0dXdd9vqai=hGuQ8kuc9pgc9s8qqaq=dirpe0xb9q8qiLsFr0=vr0=vr0dc8meaabaqaciaacaGaaeqabaqabeGadaaakeaaiiGacqWFepaDdaqhaaWcbaGaem4CamhabaGaeGOmaidaaaaa@3106@ is the variance that controls the amount of smoothing. The unstructured heterogeneity term *ui *was modelled an exchangeable normal prior, with mean zero and variance τu2
 MathType@MTEF@5@5@+=feaafiart1ev1aaatCvAUfKttLearuWrP9MDH5MBPbIqV92AaeXatLxBI9gBaebbnrfifHhDYfgasaacH8akY=wiFfYdH8Gipec8Eeeu0xXdbba9frFj0=OqFfea0dXdd9vqai=hGuQ8kuc9pgc9s8qqaq=dirpe0xb9q8qiLsFr0=vr0=vr0dc8meaabaqaciaacaGaaeqabaqabeGadaaakeaaiiGacqWFepaDdaqhaaWcbaGaemyDauhabaGaeGOmaidaaaaa@310A@. The variance components were assigned an inverse Gamma hyperprior with known hyperparameters equal to 0.001. The intercept was assumed to have a flat prior.

Estimation of the model parameters was carried out through the Markov Chain Monte Carlo (MCMC) simulation techniques as implemented in BayesX version 1.4 [20]. For our analysis we ran 35,000 iterations and discarded the initial 5,000 samples, and subsequently stored every 15th iteration, giving 2,000 samples which were summarized to get the necessary estimates. The posterior distributions of: exp(α + *u*_*i *_+ *s*_*i*_)/(1+ exp(α + *u*_*i *_+ *s*_*i*_)) were mapped, and following the approach of Kleinschmidt *et al*. [5], error margins at 2.5 and 97.5 percentiles were obtained and mapped as an approximate 95% credible interval (CI) for the posterior mean coverage. The CI also offer a good indicator of statistical power of our small area estimates.

## Results

**Figure 1:**
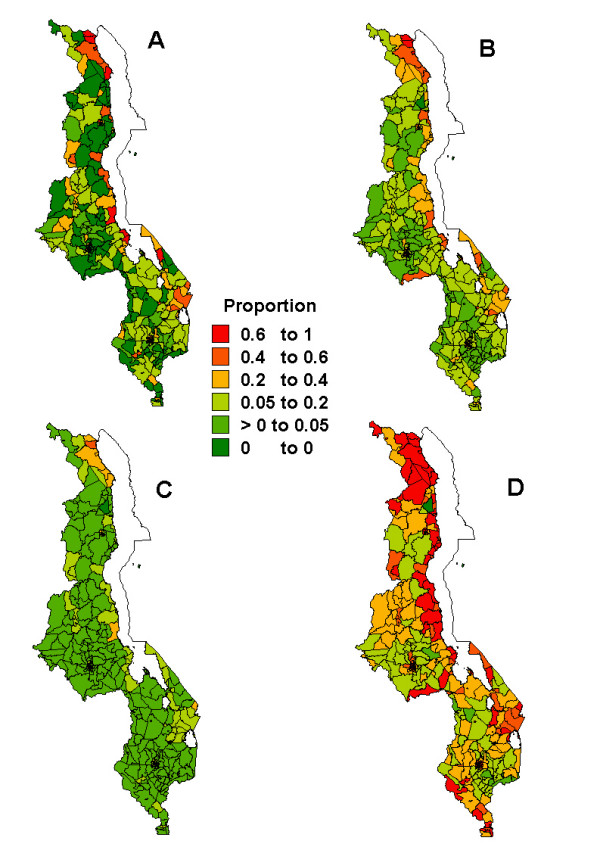
Map showing the proportion of households possessing at least one bednet at sub-district level: A) raw proportions; B) smoothed proportions; C) lower limit (p2.5) for the smoothed proportions; D) upper limit (p97.5) for the smoothed proportions.

**Figure 2:**
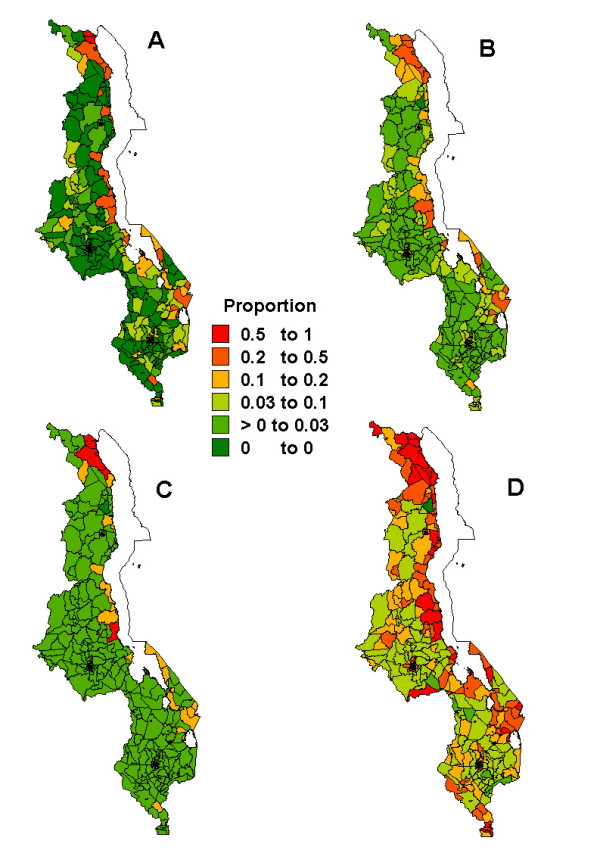
Map showing the proportion of under-five children sleeping under a bednet at sub-district level: A) raw proportions; B) smoothed proportions; C) lower limit (p2.5) for the smoothed proportions; D) upper limit (p97.5) for the smoothed proportions.

**Figure 3:**
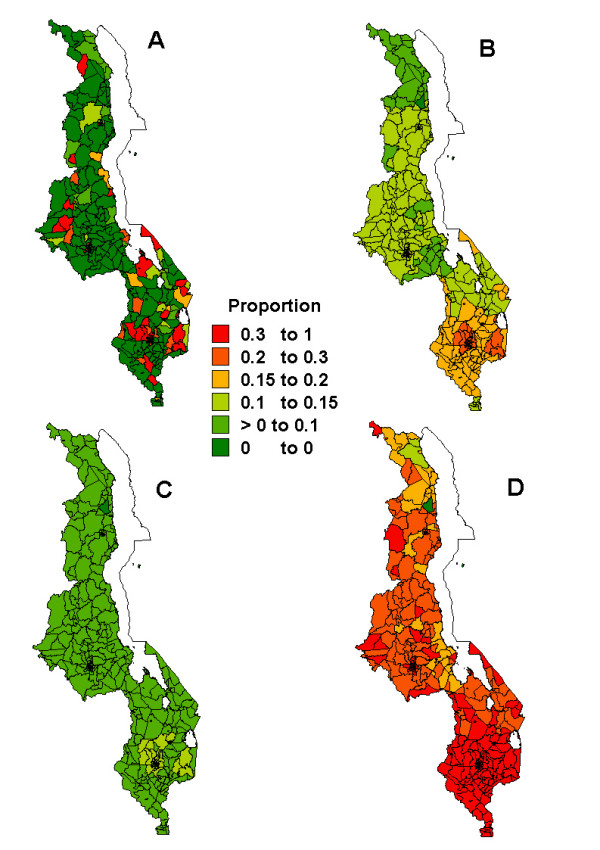
Map showing the proportion of households with bednets re-treated with insecticide within past 6–12 months at sub-district level: A) raw proportions; B) smoothed proportions; C) lower limit (p2.5) for the smoothed proportions; D) upper limit (p97.5) for the smoothed proportions.

**Figure 4:**
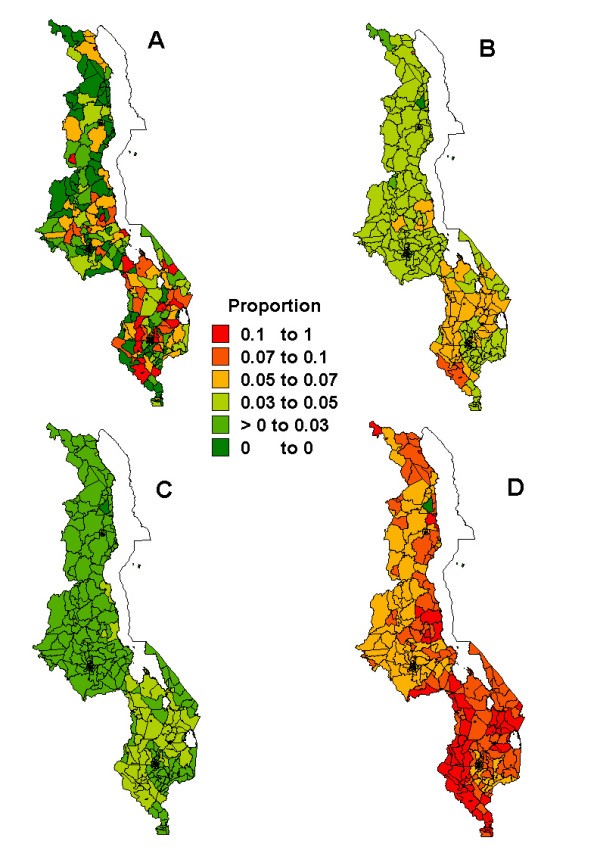
Map showing the proportion of children who received treatment for fever *<*24 hours after the onset of fever at sub-district level: A) raw proportions; B) smoothed proportions; C) lower limit (p2.5) for the smoothed proportions; D) upper limit (p97.5) for the smoothed proportions.

**Figure 5:**
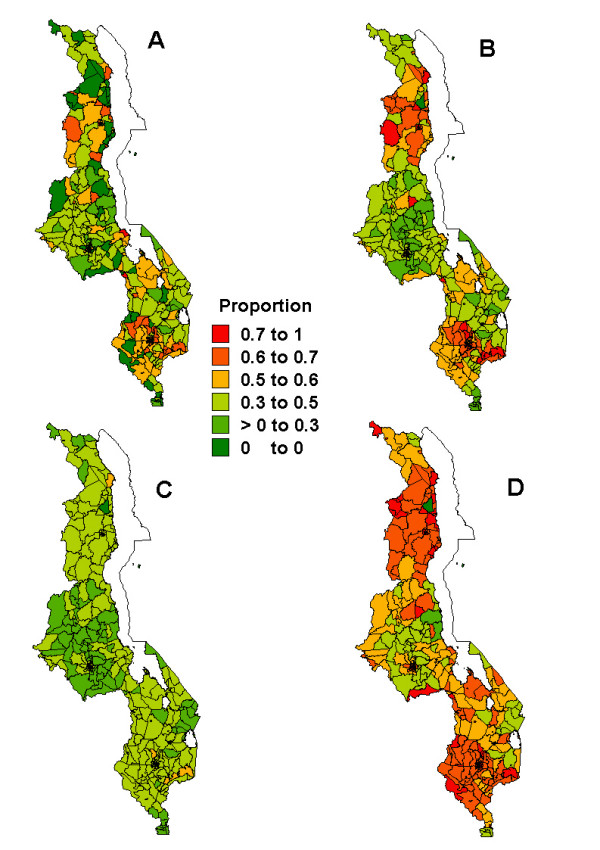
Map showing the proportion of women who received antimalarial preventive treatment during their last pregnancy at sub-district level: A) raw proportions; B) smoothed proportions; C) lower limit (p2.5) for the smoothed proportions; D) upper limit (p97.5) for the smoothed proportions.

Figures 1, 2, 3, 4, 5 present sub-district maps of the proportions of households in possession of at least one net, of children who slept under bednet the previous night, of households with nets re-treated within 6–12 months preceding the survey, of children who received anti-malarial treatment within 24 hours from the onset of fever, and of women who received antimalarial prophylaxis treatment during their last pregnancy. In each figure a map is shown of (A) the observed proportions, (B) the smoothed proportions, (C) the 2.5 percentile, and (D) the 97.5 percentile credible limits. The observed proportions for each indicator show large sampling variation, however, after smoothing patterns emerged. Our reporting below is based on the smoothed maps.

The geographical distribution of households with at least one bednet is given in Figure 1. The highest proportions of *>*40% appeared in the northern region and along the lake Malawi region. Most areas, however, were in the coverage range of 5–20%, which were predominantly in the following districts: Chikwawa, Blantyre, Mulanje, Zomba, Mangochi, Dedza, Dowa, Salima, Nkhotakota, Rumphi, Karonga and Chitipa. Compared to the raw proportions, the smoothed values were pulled towards median rate categories of between 5–20% and 20–40%. The smoothing effect was visible in districts like Rumphi, Karonga and Chitipa in the north, Dowa and Salima in the centre, and Blantyre, Mwanza and Chikwawa in the southern region. The mean bednet coverage was estimated as 19% (95% CI: 16–21%), with urban and rural areas reporting coverage of 33% (95% CI: 28–38) and 13% (95% CI: 11–16%) respectively. Similar differences were observed across regions with 30% (95% CI: 23–38%), 15% (95% CI: 12–19%), and 17% (95% CI: 140-20%) for the northern, central and southern regions respectively.

A higher proportion of children slept under a net in urban areas (18%, 95% CI: 15–22%) compared to rural areas (6%, 95% CI: 5–8%), while relatively more children slept under a net in the northern region (16%, 95% CI: 11–22%) compared to the central (7%, 95% CI: 5–10%) and southern region (9%, 95% CI: 7–11%). These disparities are evident in Figure 2. We observed that areas of highest bednet usage rates (*>*10%) were in Karonga district in the north and along the lakeshore districts of Salima and Nkhotakota in the central region, Mangochi and Machinga in the south-eastern region. We also detected isolated areas of rates of between 3–10%, particularly in Blantyre, Rumphi, Kasungu, Mchinji and Kasungu districts. Again smoothing caused coverage rates to shrink towards the median categories of 3–10% and 10–20%, with 60 of 221 areas changing categories.

Figure 3 presents the bednet re-treatment coverage. Most areas had re-treatment rates of 10–15%, and these were located in the central region and Mangochi district in the southern region. The highest re-treatment rates (*>*15%) were observed in the southern region. The northern region and Dedza district in the central region had the lowest retreatment rates (*<*10%). The national percentage coverage of households with re-treated nets was 18% (95% CI:16–19%), with 32% (95% CI: 29–35%) in urban areas and 13% (95% CI: 11–14%) in rural areas, with noticeable region differences. By examining the sub-district maps, the proportion of areas changing categories as the effect of smoothing was 81% (295 of 364 areas).

Figure 4 shows patterns of presumptive and effective case management of malaria in under-five children. There was low coverage of children receiving anti-malarials within 24 hours of onset of fever throughout the country and no differences were observed between urban and rural areas (4.8%, 95% CI: 4.5–5.1% versus 4.8%, 95% CI: 4.2–5.5% respectively), nor between regions (3.9% in the north, 4.4% in the centre and 5.4% in the south). The effect of smoothing was also evident, with 87% of all areas (317 of 364 areas) changing categories. The highest early treatment rates (*>*5%) were noted in the southern region and parts of the central region in the following districts: Salima, Dowa, Ntcheu, Mwanza, Balaka, Zomba, Chiradzulu, Chikwawa, Nsanje. However, most areas were in the 3–5% category. The lowest rates were noted at the northern tip in Chitipa district and in Kasungu district in the central region.

Intermittent prophylaxis treatment with anti-malarials during pregnancy had relatively high coverage in most areas (Figure 5), with a national coverage of 48% (95% CI: 47–50%). At regional level, coverage was 54%, 43%, and 51% for the northern, central and southern regions respectively, while urban and rural areas had coverage of 61% (95% CI: 59–62%) and 44% (95% CI: 43–46%) respectively. High intra-district rates (*>*50%) were observed in the northern and southern districts of Mzimba, Rumphi, Nkhatabay, Karonga, Chikwawa, Mwanza, Blantyre, Thyolo, Mulanje and Mangochi. Areas of low rates (*<*30%) were few and were observed in Dedza, Lilongwe, Dowa and parts of Nkhotakota districts. As shown in the maps, smoothing increased the rates in the northern region, and allowed patterns to emerge in the southern region. Within district variability was apparent, for example in Zomba, Nsanje, Mangochi, Mzimba and Mulanje districts.

## Discussion

This study demonstrates the geographical variation of antimalarial RBM indicators within districts in Malawi at a scale that is smaller than what is currently available, and emphasises the advantages of estimating coverage rates at sub-domain (subdistrict) level. The maps presented can allow generation of hypotheses that would explain the observed geographies in areas of interest. In our presentation, two maps of both raw and smoothed estimates have been shown. Raw estimates often exhibit overdispersion and sampling variation, and therefore interpretation of geographies may be difficult. The smoothed maps are presented as the solution because smoothing can normalise extreme values of those areas with much higher or lower rates than their neighbouring areas, thereby permitting patterns to emerge in the maps.

The present study indicated high coverage of bednet along the lakeshore region and Shire river highlands, which correlates with high malaria risk areas [21]. These observations support policy strategies that aim to target highly endemic areas in the country [6], which happen to be concentrated in these areas. Nevertheless, this coverage was far below the target of 60% to be achieved by the year 2006. The urban-rural divide was evident, and may reflect socio-economic differences with urban residents more likely to purchase a net. Provider factors may also contribute towards this discrepancy, for example, because nets are mostly delivered through clinics, it means rural areas with few and distant clinics are likely to have low coverage [22,23]. The observation underlines the need to consider new approaches of distributing nets [24,25]. Deliberate programmes, such as social marketing with built-in incentives for health personnel, can be used to increase bed-net coverage in rural areas [26,27], some of which are already in operation in the country through non-governmental organizations such as Population Services International, Canadian Physicians Aid and Relief, and Blantyre Malaria Initiative [22,25,28]. Nets distribution can be accelerated through other health promotion campaigns for instance during immunization operations. Community based ITN committees have also been promoted as means of markedly increasing coverage [6]. These programmes should target critically poor areas, with emphasis in highly malarious locales of the country [21,27,28].

The small proportion of children using nets may be a factor associated with limited coverage of bednet ownership. Macintyre *et al*. [23] found that, in Eritrea, ITN use was a factor of number of ITNs in the household. Households with high net-to-person ratios were more likely to use bednet during and following the rainy season. Reports also establish that high levels of usage can be maintained, even in the absence of programmes promoting their utility [24,29]. Similar to bednet usage, low coverage was observed for bednet re-treatment, and this finding is consistent with rates in most malaria-endemic sub- Saharan African countries [13]. Relatively high usage and re-treatment coverage in urban areas seems to suggest that urban residents are well informed about benefits of bednet use and re-treatment. In rural areas, economic factors affect rates of re-treatment and seasonal variation in cash incomes may impact on the ability to pay for re-treatment [29].

It is evident that bednet ownership alone is a poor indicator of malaria control, and despite good distribution points in the country [25], does not translate into use and retreatment. Yet, usage and re-treatment are important indicators in the RBM campaign because these prevent contact with biting mosquitoes, and hence are critical to reducing infection and interruption of transmission. The wide gap detected between the geographical pattern of net distribution with highest coverage, for instance along lake Malawi (Figure 1) and of re-treatment, highest coverage being in the south of the country (Figure 3), suggests that concerted effort need to be taken, particularly in high malaria risk areas, to change perceptions about malaria and protection from malaria. Both bednet use and retreatment should be enhanced through regular health promotion campaigns. Community meeting with the aim of stressing the importance of re-treatment may be appropriate to stimulate the demand for re-treatment [29]. Establishing outlets for re-treatment closer to people's homes may also promote high and sustainable levels of re-treatment. The low re-treatment coverage rates in the country and the region underscore the need for long lasting ITNs as a feasible, long-term malaria tool. Indoor residual spraying and other vector control measures are effective alternatives or supplement to ITNs [30].

Patterns identified on coverage of PECM (Figure 4) reveal that clusters of relatively high coverage (*>*10%) were only in the south-western districts of Chikwawa and Mwanza, with the rest of the country indicating low coverage. The geographical pattern of PECM might be due to a combination of provider compliance or coverage differences. Stock-outs of antimalarials at health facilities would entail similar geographical differences in PECM coverage for children who received antimalarials from clinics, while inaccessibility and unavailability of health facilities may hamper early diagnosis, hence lower PECM coverage. Low coverage may also be influenced by treatment seeking behaviour. Generally, most fever treatments occur outside the formal sector, because of, among other things, unavailability of transport and long travel distance to health facilities, poor assessment of severity of the disease, and traditional beliefs that fevers can be treated by home remedies [6,32], thereby missing an opportunity of early diagnosis and treatment.

Compared to surveys carried out in 1994, which reported about 80% of children receiving prompt treatment [31], the differences are substantial, and considering that self treatment is pervasive, greater effort is required to improve access, quality and coverage of home case management or timely and effective care at health facilities in the country. Introducing and training community shopkeepers and community health workers in areas of low coverage might improve uptake coverage of PECM [33]. Information, education and communication (IEC) health campaigns should first understand and build on the patterns of treatment seeking behaviour to promote good treatment practices and rational drug use.

Maps of IPTp (Figure 5) show that a high proportion of women received antimalarial prophylaxis during their last pregnancy, although most coverage rates fell below the target level of 60%. The striking geographical patterns may also reflect spatial differences in provider compliance, coverage or both. The presence of relatively few clinics in rural areas compared to urban areas, requiring pregnant women to travel long distances to attend the nearest facility, may impact IPTp coverage in such areas [15,27]. Despite increased antenatal attendance over time [34-36], late presentation, confusion by nurses about the timing of the two doses of sulphadoxine-pyrimethamine (SP) [6], and stock-outs of SP at antenatal care clinics [26,36], may also contribute to low coverage rates, for example in urban areas of Lilongwe and Mzuzu.

The task of increasing coverage in these interventions is enormous, and requires operational strategies on implementation. It is very likely though that the coverage has changed since the year 2000. An important development since the 2000 MDHS survey was the implementation of the 2004 MDHS, a fourth in a series of surveys in the country. The data are being processed, and these would allow assessment of changes in coverage of which our maps are the baseline for comparison at small area level. In addition, in 2003, the health management information system introduced new key performance indicators, including ITN possession, ITN use and IPTp coverage. Coverage of these indicators are being reported on quarterly basis from all health facilities in the country. Because of the extensive reporting, the data combined with small area analysis would allow patterns overstepped by our analysis to emerge, enhancing community health assessment and planning.

This study has shown that geographical smoothing stabilises estimates and allows patterns to emerge, however, these have limitations because the procedure may over-smooth estimates in some areas, such that real differences between adjacent areas may disappear. Nevertheless, the published CI maps should help with the interpretation of the results. The validity of the estimates can be evaluated. Jia *et al*. [1] proposed a number of discrepancy statistics for validating the sub-domain estimates, for example, aggregated domain (district) average from sub-domain (sub-district) estimates can be compared against domain composite estimates (which more accurately reflects the true domain rates) using Pearson's correlation coefficient or the root of mean square errors.

Smoothing also has an effect of areas changing categories, and often areas of extreme values are pulled towards the median. It should be noticed that when data are not sparse, spatial smoothing would help less. This is apparent in the pattern of net coverage (Figure 1) where the proportions and denominators were relatively higher than in the other outcomes, and the number of areas changing categories were evidently few, compared to those for bednet retreatment and PECM (Figures 3 and 4).

Our analysis highlighted within-country variation in DHS malaria indicators, and according to our knowledge, is the first in Malawi. Although comparable examples exist elsewhere [37,38], these are at regional level. The increasing emphasis of public health policy on local, or community health assessment and planning, implies that such regional maps are not useful. Since the DHS apply standard methodology, and the sample sizes are comparable to that in other malaria-endemic African countries, exactly the same small area analyses can be carried out to allow an appraisal of within-country variation and facilitate cross-country comparison at relevant small area levels.

Although the Malawi DHS provides useful data that can be used to identify gaps in malaria control, a potential source of bias is that the survey was conducted in summer, a time during which malaria transmission is low. This is likely to downward bias the observed usage of bednets as people will resort to using nets only when there is a perceived risk. Nevertheless, these biases may not vary geographically, hence, the pattern of usage can still be established.

The multi-stage sampling scheme employed by the DHS, while reducing the survey's travel costs and achieving a nationally representative sample of many households in some EAs but no households in other EAS, results in samples that have little dispersion. This constraint in geographical spread may bias the spatial pattern exhibited in the maps. Although not carried out here, the mean sub-district level estimates should be evaluated to see how close these are to the national estimates. Realistically, improved estimates can be obtained by drawing a large representative sample in each district to obtain adequate sample points at sub-domains. Because this may not be cost-effective, our methodology bridges the need for statistically sufficient statistical power and the lack of sufficient data to provide accurate design-based estimates of RBM indicators coverage.

Against these limitations, the maps presented here suffice for the purpose of identifying clusters of persistently low population coverage of core RBM indicators, and this may be of significance to policy makers and implementors in Malawi. Although the techniques of producing these maps are sophisticated, various researchers are using geographical maps in which health outcomes and programme indicators are visualised as means of presenting their research results [3-5]. These maps, therefore, provide much needed evidence-based information which may serve as a baseline to monitor and evaluate progress towards RBM goals in Malawi.

## Competing interests

The author(s) declare that they have no competing interests.

## Authors' contributions

LNK conceptualised, analysed and drafted the manuscript. CCA and IK participated in the conception, and critical review of the manuscript. All authors read and approved the final manuscript.
